# Dose-response associations of triglyceride to high-density lipoprotein cholesterol ratio and triglyceride–glucose index with arterial stiffness risk

**DOI:** 10.1186/s12944-024-02095-z

**Published:** 2024-04-20

**Authors:** Wenkai Zhang, Weifeng Huo, Huifang Hu, Tianze Li, Lijun Yuan, Jinli Zhang, Yifei Feng, Yuying Wu, Xueru Fu, Yamin Ke, Mengmeng Wang, Longkang Wang, Yaobing Chen, Yajuan Gao, Xi Li, Liang Sun, Jinyuan Pang, Zeqiang Zheng, Fulan Hu, Ming Zhang, Yu Liu, Dongsheng Hu, Yang Zhao

**Affiliations:** 1 Department of Epidemiology and Health Statistics, College of Public Health, Zhengzhou University, Zhengzhou, Henan People’s Republic of China; 2 Department of Social Medicine and Health Management, College of Public Health, Zhengzhou University, Zhengzhou, Henan 450001 People’s Republic of China; 3 Department of Preventive Medicine, School of Public Health, Shenzhen University Health Science Center, Shenzhen, Guangdong People’s Republic of China; 4 Department of Biostatistics and Epidemiology, school of Public Health, Shenzhen University Health Science Center, Shenzhen, Guangdong People’s Republic of China; 5 Guangdong provincial Key Laboratory of Regional Immunity and Diseases, Shenzhen University Health Science Center, Shenzhen, Guangdong People’s Republic of China; 6 Department of General Practice, The Affiliated Luohu Hospital of Shenzhen University Health Science Center, Shenzhen, Guangdong People’s Republic of China; 7 Department of Epidemiology and Biostatistics, College of Public Health, Zhengzhou University, 100 Kexue Avenue, Zhengzhou, Henan 450001 People’s Republic of China

**Keywords:** Triglyceride to high-density lipoprotein cholesterol ratio, Triglyceride-glucose index, Arterial stiffness, Pulse wave velocity, Meta-analysis

## Abstract

**Background:**

The triglyceride to high-density lipoprotein cholesterol (TG/HDL-C) ratio and triglyceride-glucose (TyG) index are novel indexes for insulin resistance (IR). We aimed to evaluate associations of TG/HDL-C and TyG with arterial stiffness risk.

**Methods:**

We enrolled 1979 participants from the Rural Chinese Cohort Study, examining arterial stiffness by brachial-ankle pulse wave velocity (baPWV). Logistic and linear regression models were employed to calculate effect estimates. For meta-analysis, we searched relevant articles from PubMed, Embase and Web of Science up to August 26, 2023. The fixed-effects or random-effects models were used to calculate the pooled estimates. We evaluated dose-response associations using restricted cubic splines.

**Results:**

For cross-sectional studies, the adjusted ORs (95%CIs) for arterial stiffness were 1.12 (1.01–1.23) and 1.78 (1.38–2.30) for per 1 unit increment in TG/HDL-C and TyG. In the meta-analysis, the pooled ORs (95% CIs) were 1.26 (1.14–1.39) and 1.57 (1.36–1.82) for per 1 unit increment of TG/HDL-C and TyG. Additionally, both TG/HDL-C and TyG were positively related to PWV, with β of 0.09 (95% CI 0.04–0.14) and 0.57 (95% CI 0.35–0.78) m/s. We also found linear associations of TG/HDL-C and TyG with arterial stiffness risk.

**Conclusions:**

High TG/HDL-C and TyG were related to increased arterial stiffness risk, indicating TG/HDL-C and TyG may be convincing predictors of arterial stiffness.

**Supplementary Information:**

The online version contains supplementary material available at 10.1186/s12944-024-02095-z.

## Introduction

Cardiovascular diseases (CVDs), particularly stroke and ischemic heart disease (IHD) [1], constitute the top-ranking causes of premature death globally, and are the main contributors to the global burden of health [2]. Arterial stiffness, occurring with aging and a variety of pathological conditions, not only has a profound impact on the cardiovascular system but contributes significantly to the worldwide burden of CVDs [3]. In particular, arterial stiffness has been widely acknowledged as a valuable biomarker for identifying populations at higher risk of CVDs and mortality [4–7]. Given the genesis of arterial stiffness as a long-term pathological process, early identification of subjects at increased arterial stiffness risk and intervention with appropriate preventive strategies are both crucial to reducing the global health burden.

Insulin resistance (IR) is generally defined as reduced sensitivity to physiological insulin levels in insulin-targeting tissues [8]. It is likely to result in arterial stiffness by promoting dyslipidemia, a pro-inflammatory state, and causing endothelial damage [9–11]. Classically, the hyper-insulinemic euglycemic clamp is widely accepted as the gold standard for determining IR, but it is laborious, high-priced, and time-consuming [12], limiting its applicability in clinical settings. Interestingly, the triglyceride to high-density lipoprotein cholesterol ratio (TG/HDL-C) and triglyceride-glucose index (TyG), derived from fasting plasmsa glucose (FPG) and triglycerides [13, 14], have been recognized as convincing indictors of IR. Unfortunately, the relationships of TG/HDL-C and TyG with arterial stiffness risk are still being debated. Despite previous studies having evaluated associations of TG/HDL-C and TyG with arterial stiffness risk [15–17], conclusions were inconsistent across studies. Several studies reported positive relationships between TG/HDL-C and TyG and arterial stiffness risk [15, 17–19], but the associations disappeared in other studies [16, 20–22]. A review published in 2020 argued that TG/HDL-C and TyG may be accurate indicators of arterial stiffness [23], but it failed to quantitatively clarify the associations.

This study therefore aimed to explore the relationships of TG/HDL-C and TyG with arterial stiffness risk among rural Chinese adults and to summarize the dose-response associations of TG/HDL-C and TyG with arterial stiffness.

## Methods

### Cross-sectional study

#### Study design and population

This study was based on the Rural Chinese Cohort Study (RCCS) which was conducted to assess disease patterns and risk factors for non-communicable diseases in the rural population [24]. In all, 17,641 participants were re-examined in the second follow-up survey from July to August in 2018–2019. Among these, we enrolled a total of 2092 subjects who underwent an examination for arterial stiffness. Participants with missing data on TG, HDL-C, or FPG were excluded (*n* = 103). We further excluded participants with bilateral ankle-brachial indexes (ABIs) < 0.9 (*n* = 10). Ultimately, 1979 individuals were included in the current analysis **(Supplemental Fig. 1)**.

In addition, we calculated the minimum sample size based on the proportion of participants with arterial stiffness (p) of 16.51% and the standard normal variable (Z_1−α/2_) of 1.96 [25]. Due to the 10% non-response response, the final sample size was 1471. This study therefore enrolled a sufficient number of participants.

#### Data collection and definition

Socio-demographic data (age, gender, education level, and marital status), lifestyle information (physical activity, smoking, and alcohol consumption), and medical records were obtained using structured questionnaires in in-person interviews. In physical examinations, we measured participants’ weight, height, waist circumference (WC), and blood pressure (BP) according to established and standardized procedures, as previously described [24]. Body mass index (BMI) was defined as weight divided by height squared (Kg/m^2^). Systolic and diastolic blood pressure (SBP and DBP) were assessed using an electronic sphygmomanometer (HEM-770AFuzzy, Omron). Overnight fasting blood samples were collected to measure biochemical parameters, including the levels of FPG, total cholesterol (TC), TG, HDL-C, and low-density lipoprotein cholesterol (LDL-C) using an automatic biochemical analyzer (HITACHI, model 7060, Tokyo). TG/HDL-C and TyG were defined as TG (mmol/L) divided by HDL-C (mmol/L) and Ln [fasting TG (mg/dL) *FPG (mg/dL)/2], respectively [26].

#### Ascertainment of arterial stiffness

The baPWV and ABI were tested simultaneously by trained investigators using an arteriosclerosis device (BP-203 RPE III, Omron). After participants had been resting for over 5 min in the supine position, the pressure waveform and transmission distance bilateral brachial and ankle arteries were automatically recorded by 4 cuffs with volume pulse and oscillating pressure sensors. We calculated baPWV values by the formula (La-Lb)/Tba. La and Lb are defined as the length from the heart to the ankle and brachium, and Tba is the transit time between the ankle and brachial waveforms. Accordingly, ABI was determined as ankle SBP divided by brachial SBP. As the reliability of baPWV values may be diminished in participants with severe atherosclerosis [27], we excluded participants with bilateral ABIs < 0.9. The contralateral baPWV was taken if individuals had unilateral ABI < 0.9, and the higher baPWV values of the right and left sides were considered if no ABI < 0.9. Additionally, we defined arterial stiffness as baPWV value ≥ 1800 cm/s [28].

#### Statistical analysis

The characteristics of study populations were described as frequency (percentage) for categorical variables and median (interquartile range) for continuous variables. The differences in participants without or with arterial stiffness were compared by the Kruskal-Wallis or χ^2^ test.

Logistic regression was used to assess relationships of TG/HDL-C and TyG with arterial stiffness, while linear trends were assessed by applying the median values of quartiles as continuous variables. Linear regression was used to assess associations of TG/HDL-C and TyG with baPWV levels, with beta coefficient (β) and 95% CI. Given possible confounders, we examined three models: model 1 included age and gender; model 2 included model 1 plus marital status, education level, smoking, alcohol drinking, and physical activity; and model 3 included model 2 plus BMI, WC, SBP, DBP. Subgroup analyses were conducted for age (< 65 and ≥ 65 years), sex (men and women), smoking (never and ever/current), alcohol drinking (no and yes), and BMI (< 24 and ≥ 24 Kg/m^2^). We further explored the interaction effects of blood pressure and fasting blood glucose levels with TG/HDL-C and TyG for arterial stiffness. Sensitivity analyses were conducted with the standard for arterial stiffness of 1400 cm/s. Additionally, we explored the relationships between TG/HDL-C and TyG with healthy vascular aging (HVA), normal vascular aging (NVA), and early vascular aging (EVA), defined as < baPWV-percentile < 10th, 10th ≤ baPWV-percentile ≤ 90th, and baPWV-percentile > 90th of the population stratified by age and sex [29].

SAS v9.4 was taken for all analyses (SAS Institute Inc., Cary, NC, USA), with a two-sided *P* value < 0.05 considered statistically significant.

### Dose-response meta-analysis

#### Search strategy

We comprehensively searched PubMed, EMBASE and Web of Science up to August 26, 2023 for all relevant records in the English-language, using combinations of MeSH and free-text terms. Detailed search strategies are shown in **Supplemental Table 5**. Extra eligible publications were manually screened for the bibliographical references.

This dose-response meta-analysis was performed according to the Preferred Reporting Items for Systematic Reviews and Meta-Analyses (PRISMA) [30]. The registration number on the International Prospective Register of Systematic Reviews (PROSPERO) is CRD42022325395.

#### Study selection

We included studies if: (1) they were cross-sectional, case-control, or cohort studies; (2) their populations were aged ≥ 18; (3) the exposure included TG/HDL-C or TyG index; (4) the outcome included arterial stiffness (defined as PWV) or PWV levels; and (4) they provided quantitative estimates and 95% CIs or standard errors (or relevant data to compute these). If multiple publications derived from the same study, the included data was taken from studies with the largest sample size or the most informative report. Reviews, editorials, and letters were excluded. In addition, two authors (W.Z. and Y.K.) separately searched relevant literature, reviewed titles, and abstracts, and screened full texts using the same selection criteria.

#### Data extraction and quality assessment

Two authors (W.Z. and Y.K.) separately collected information regarding first author, publication year, country, study design, sex, age, study size, number of cases, follow-up years if cohort studies, measurement of exposure, assessment of outcome, confounding factors, and most adjusted estimates (ORs, risk ratios [RRs], hazard ratios [HRs], or β) with 95% CIs. If a study presented both central (carotid-femoral or aortic pulse wave velocity [cfPWV or aPWV]) and peripheral measures (baPWV), we extracted results of central measures, in accordance with the gold standard approach for evaluating arterial stiffness [31].

The Agency for Healthcare Research and Quality scale (AHRQ), including 11 aspects with three answers (0, no or unclear; 1, yes), was taken to evaluate the quality of cross-sectional studies [32]. Studies ware categorized as poor (0–3), general (4–7), or high quality (8–11), respectively [33]. The Newcastle-Ottawa Scale (NOS) was applied to assess the quality of cohort studies with scores ranging from 0 to 9 across 8 items [34]. Any uncertainty was resolved in consultation with the third reviewer (D.H.).

#### Data synthesis and analysis

Our effect estimates incorporated both binary data (arterial stiffness) and continuous data (PWV levels) in this meta-analysis. ORs and β values with corresponding 95% CIs were taken as general effect measurements for associations of TG/HDL-C and TyG with arterial stiffness risk and PWV levels. We assumed that the RRs/HRs given in eligible records were about equal to ORs [35]. Any results in original articles that stratified by different gender or exposure types were regarded as independent studies. If the number of cases in each group was not published, we determined it via the provided RRs/HRs and the count of total cases [36]. When data on exposed subjects or person-years in a category was missing, we assumed it to be equal in size [36]. The midpoint was taken as the mean TG/HDL-C or TyG if the exposure category was reported as a range [36]. If the lowest or highest category was open-ended, the range was assumed to be the same width as the nearest group, and the midpoint was determined accordingly [37].

Heterogeneity was evaluated through Cochran’s Q and *I*^*2*^ statistics [38]. For the Q statistic, *P* < 0.1 was considered as statistical significance. Regarding *I*^*2*^ statistics, *I*^*2*^ values of 75%, 50%, and 25% were taken to be high, general, and poor heterogeneity, respectively [38]. Fixed-effects models were selected to pool ORs or β values with 95% CIs if *I*^*2*^ < 50%; otherwise, random-effects models were applied. Further, we conducted generalized least-squares regression to evaluate dose-response relationships [39]. We also explored potential linear or nonlinear trends through modelling TG/HDL-C and TyG with restricted cubic splines, with 3 knots located at the 75th, 50th, and 25th percentiles of distribution [40]. Only studies with at least 3 categories of TG/HDL-C or TyG were included in the dose-response analysis.

Subgroup analyses were performed between sex (men, women, and both), average age (≤ 50 and > 50 years), region (Asia, and Europe/United States [US]), sample size (≤ 1000 and > 1000), study design (cross-sectional and cohort studies), study quality (high and medium quality), and PWV assessment site (baPWV, cfPWV, and aPWV), then adjusted for some major potential confounders (BMI, smoking, alcohol drinking, FPG, and SBP) in the study-specific dose-response analysis. Moreover, we conducted meta-regression analyses among different subgroups [41], and performed sensitivity analyses through excluding one study at a time. If there were 8 or more available studies, publication bias (small-study effect) was examined with the Egger’s test and funnel plots [42]. The trim and fill method was employed for adjustment when publication bias was found.

Stata 14.0 was taken for all analyses (Stata Corp, College Station, TX, USA). Statistical significance was defined as two-sided *P* < 0.05.

## Results

### Cross-sectional study

The characteristics of our study’s populations, without and with arterial stiffness, are shown in Table 1. Significant differences were found in the distributions of age, marital status, alcohol consumption, physical activity, BMI, SBP, DBP, FPG, TG, TyG index, and baPWV (all *P* < 0.05).

**Table 1:** Baseline characteristics of study participants with and without Arterial stiffness

characteristics	Overall (*N* = 1,949)	Arterial stiffness (*N* = 414)	Non-arterial stiffness (*N* = 1,565)	P
Age, years	62 (53–69)	70 (64–75)	58 (51–66)	< 0.001
Men, n (%)	716 (36.18)	151 (36.47)	565 (36.10)	0.889
Married or cohabitating, n (%)	1690 (86.80)	306 (75.74)	1384 (89.70)	< 0.001
High school or above, n (%)	187 (9.45)	29 (7.00)	158 (10.10)	0.056
Smoking, n (%)	534 (27.38)	110 (27.23)	424 (27.43)	0.937
Alcohol drinking, n (%)	216 (11.11)	24 (5.94)	192 (12.46)	< 0.001
Physical activity, MET·min/week	7461.27 (4213.09-12507.85)	5040.00 (2741.54-7810.58)	8234.12 (4826.92-13525.19)	< 0.001
BMI, Kg/m^2^	25.27 (22.77–27.72)	25.02 (22.75–27.24)	25.33 (22.80-27.83)	0.043
WC, cm	87.55 (80.20-95.05)	88.70 (81.55–95.40)	87.40 (80.05–94.90)	0.145
SBP (mmHg)	124.67 (113.50-138.83)	141.17 (129.00-152.83)	121.33 (111.33-133.33)	< 0.001
DBP (mmHg)	74.67 (67.67–81.67)	78.67 (70.50-85.67)	73.33 (67.00-80.67)	< 0.001
FPG (mmol/L)	5.41 (5.09–5.99)	5.59 (5.12–6.38)	5.39 (5.09–5.93)	< 0.001
TC (mmol/L)	4.37 (3.80–4.98)	4.38 (3.77–5.15)	4.36 (3.80–4.95)	0.297
TG (mmol/L)	1.49 (1.08–2.14)	1.53 (1.12–2.28)	1.46 (1.07–2.12)	0.026
HDL-C (mmol/L)	1.32 (1.11–1.55)	1.32 (1.12–1.54)	1.32 (1.11–1.55)	0.626
LDL-C (mmol/L)	2.41 (1.98–2.88)	2.40 (1.94-3.00)	2.41 (1.99–2.85)	0.645
TG/HDL-C	1.11 (0.73–1.83)	1.17 (0.77–1.88)	1.08 (0.72–1.82)	0.158
TyG index	8.81 (8.45–9.23)	8.93 (8.53–9.32)	8.78 (8.43–9.20)	< 0.001
baPWV (m/s)	15.19 (13.28–17.48.)	19.82 (18.87–21.32)	14.36 (12.92–15.86)	< 0.001

Note: Data are presented as median (Q1-Q3) or number (%). BMI, body mass index; WC, waist circumference; SBP, systolic blood pressure; DBP, diastolic blood pressure; FPG, fasting plasma glucose; TC, total cholesterol; TG, triglycerides; HDL-C, high-density lipoprotein cholesterol; LDL-C, low-density lipoprotein cholesterol; TyG, triglyceride-glucose; baPWV, brachial-ankle pulse wave velocity

**Fig. 1:**
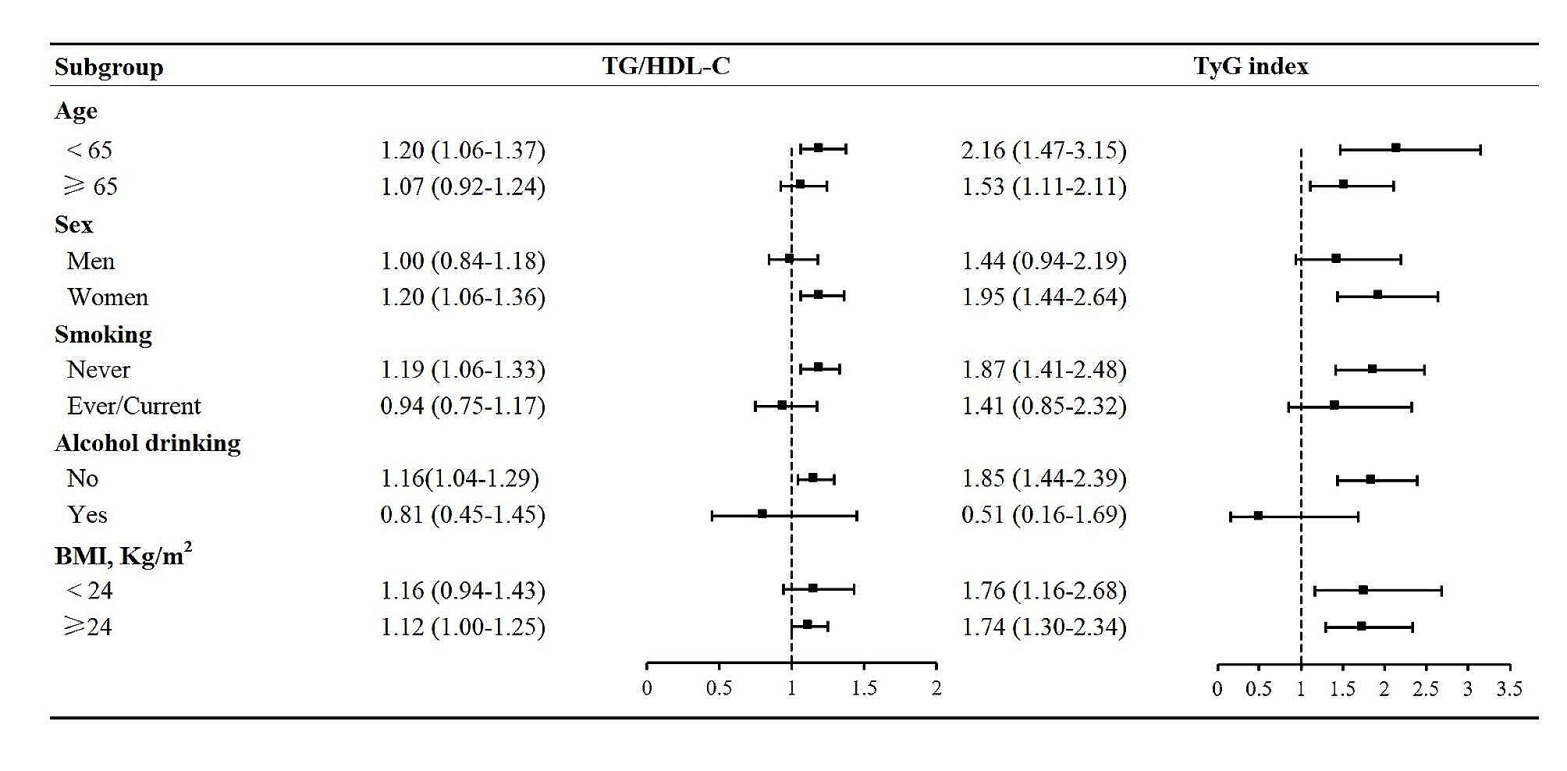
Subgroup analysis of associations between TG/HDL-C and TyG index (per 1 unit increment) and arterial stiffness Data are shown as OR (95% CI). Note: Adjusted for age, gender, marital status, education, smoking, alcohol drinking, physical activity, body mass index, waist circumference, systolic and diastolic blood pressure, total cholesterol, a for TG/HDL-C, b for TyG index

We further observed positive and linear associations of TG/HDL-C and TyG with arterial stiffness in the present study (Table 2**)**. In the multivariable adjusted model 3, the ORs (95% CIs) for arterial stiffness across TG/HDL-C quartiles 1, 2, 3, 4 were 1.00 (reference), 1.43 (0.95–2.15), 1.70 (1.13–2.55), and 1.81 (1.18–2.78) (*P*_trend_ = 0.005), respectively, while the ORs (95% CIs) for arterial stiffness were 1.00 (reference), 1.57 (1.04–2.37), 1.91 (1.25–2.92), and 2.73 (1.75–4.26) across TyG quartiles (*P*_trend_ < 0.001), respectively. Similarly, the ORs (95% CIs) for arterial stiffness with per 1 unit increment in TG/HDL-C and TyG were 1.12 (1.01–1.23) and 1.78 (1.38–2.30) in model 3, respectively. Additionally, multivariable linear regressions illustrated that per 1 unit increment in TG/HDL-C and TyG were related to 0.11 (0.03–0.19) and 0.58 (0.39–0.79) m/s increase in baPWV. When subjects were stratified by potential risk factors, consistent results were observed (Fig. 1). As shown in **Supplemental Table 2**, no significant interaction was discovered between TG/HDL-C and arterial stiffness (all *P*_interaction_ > 0.05), but we found a significant interaction between SBP and TyG for arterial stiffness (*P*_interaction_ = 0.025).

**Table 2:** Associations of TG/HDL-C and TyG index with arterial stiffness (baPWV ≥ 1800 cm/s) and baPWV levels

	Range	Model 1	Model 2	Model 3
TG/HDL-C				
Q1	< 0.73	Ref	Ref	Ref
Q2	0.73–1.11	1.20 (0.84–1.70)	1.22 (0.86–1.75)	1.43 (0.95–2.15)
Q3	1.11–1.83	1.60 (1.14–2.25)	1.54 (1.09–2.19)	1.70 (1.13–2.55)
Q4	≥ 1.83	1.66 (1.17–2.35)	1.64 (1.15–2.34)	1.81 (1.18–2.78)
*P* for trend		0.001	0.003	0.005
Per 1 unit increase		1.11 (1.02–1.21)	1.11 (1.02–1.20)	1.12 (1.01–1.23)
Per 1 unit increase, m/s*		0.13 (0.04–0.21)	0.12 (0.03–0.20)	0.11 (0.03–0.19)
**TyG index**				
Q1	< 8.45	Ref	Ref	Ref
Q2	8.45–8.81	1.18 (0.82–1.69)	1.27 (0.88–1.83)	1.57 (1.04–2.37)
Q3	8.81–9.23	1.63 (1.15–2.32)	1.68 (1.17–2.41)	1.91 (1.25–2.92)
Q4	≥ 9.23	2.14 (1.50–3.03)	2.22 (1.55–3.18)	2.73 (1.75–4.26)
*P* for trend		< 0.001	< 0.001	< 0.001
Per 1 unit increase		1.64 (1.34–2.01)	1.64 (1.33–2.02)	1.78 (1.38–2.70)
Per 1 unit increase, m/s*		0.65 (0.45–0.85)	0.64 (0.44–0.84)	0.58 (0.39–0.79)

Data are odds ratio (ORs) or beta coefficient (β) and 95% confidence intervals (95% CIs).

Model 1: adjusted for age and gender

Model 2: adjusted for age, gender, marital status, education, smoking, alcohol drinking, physical activity

Model 3: adjusted for age, gender, marital status, education, smoking, alcohol drinking, physical activity, body mass index, waist circumference, systolic blood pressure, diastolic blood pressure, total cholesterol

*: multiple linear regressions for PWV levels according to TG/HDL-C or TyG index (per 1 unit increment)

In addition, with the standard for arterial stiffness of 1400 cm/s, the results were consistent with the main results (**Supplemental Table 1**). The relationships of TG/HDL-C and TyG with HVA, NVA and EVA were similar to those of arterial stiffness (**Supplemental Tables 3 and 4)**.

### Dose-response meta-analysis

#### Literature search and study characteristics

Our initial search found 6343 relevant records. Of those, 22 articles (comprising 40 studies) were selected for meta-analysis [14–22, 25, 29, 43–53]. Finally, this meta-analysis included 41 eligible studies, including the current study. The selection and exclusion details are illustrated in a flow diagram (**Supplemental Fig. 2**).

Details of included articles are provided in **Supplemental Table 5**. Overall, we included 18 cross-sectional and 5 cohort study articles, comprising 66,676 individuals. Of those, 38 studies assessed the associations between surrogate estimates of IR (16 for TG/HDL-C and 22 for TyG) and arterial stiffness risk [15–22, 25, 29, 43–46, 48–52], while 15 examined the associations of surrogate estimates of IR with PWV levels [14, 17, 22, 25, 29, 47, 49, 51, 53], including 5 for TG/HDL-C and 10 for TyG. The mean AHRQ of cross-sectional studies was 7.8 (**Supplemental Table 7**), and the average NOS score of cohort studies was 8.4 (**Supplemental Table 8**).

#### TG/HDL-C and arterial stiffness risk

**Fig. 2:**
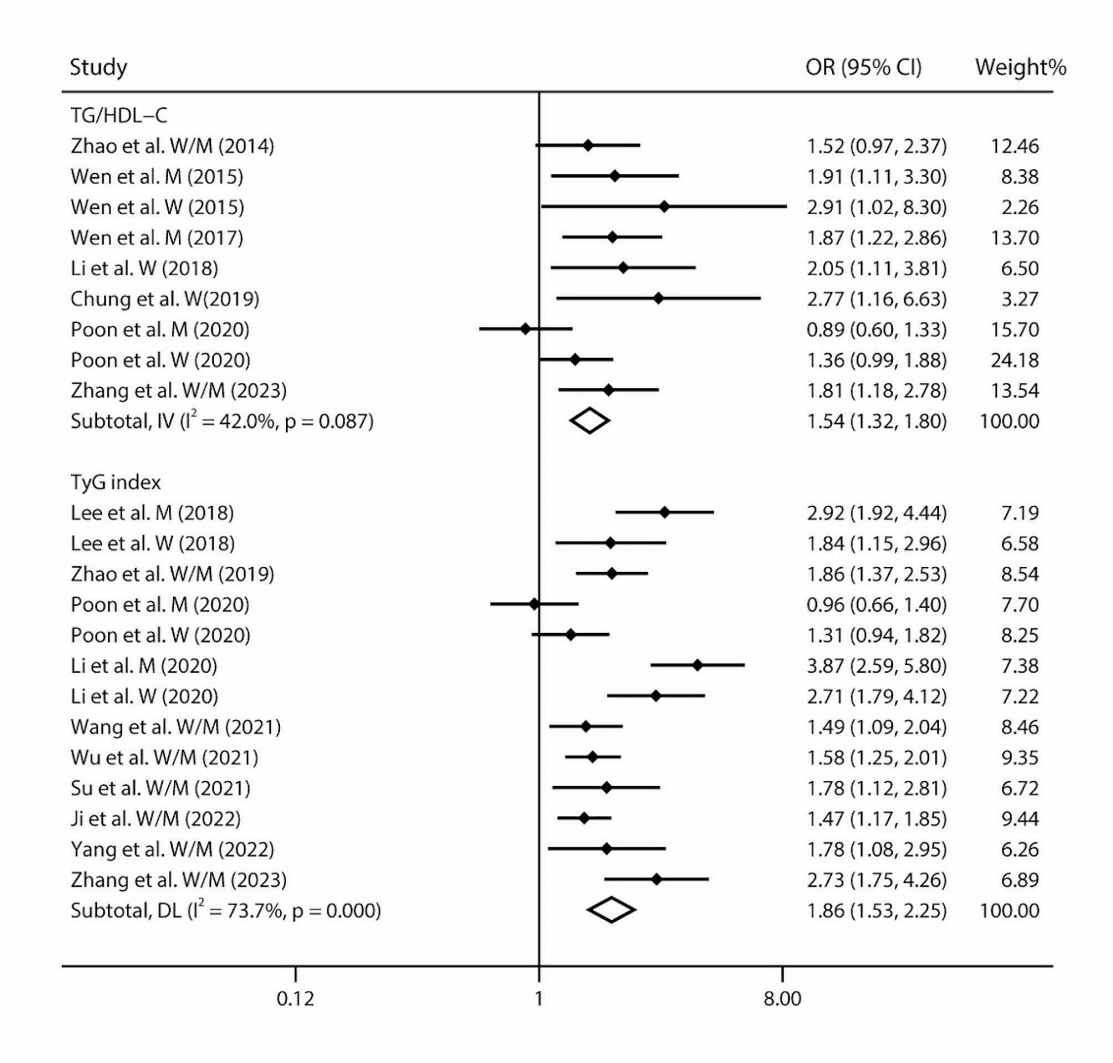
Forest plot of study-specific risks for arterial stiffness with highest versus lowest TG/HDL-C and TyG index

Our meta-analysis included 9 studies exploring the correlation of arterial stiffness with the highest versus lowest TG/HDL-C levels [15, 16, 20, 43, 44]. The summary OR of the highest group was 1.54 (95% CI 1.32–1.80, *I*^2^ = 42.0%, *P*_heterogeneity_ = 0.087; Fig. 2). When conducting the sensitivity analysis, the pooled OR was robust. No publication bias was revealed by the Egger’s tests (*P* = 0.051) and funnel plots (**Supplemental Fig. 3A**).

**Fig. 3:**
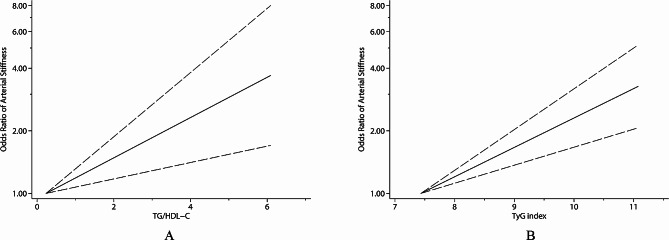
Dose?response association of TG/HDL-C (**A**) and TyG index (**B**) with the risk of arterial stiffness.

**Fig. 4:**
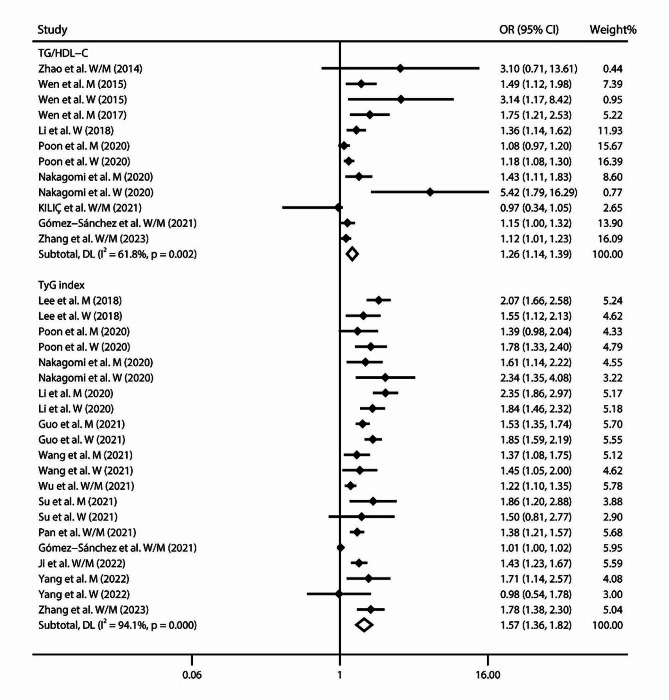
Forest plot of study-specific risks between TG/HDL-C and TyG index (per 1 unit increment) and arterial stiffness.

Moreover, we discovered a positive linear relationship between TG/HDL-C and arterial stiffness risk [15, 16, 19–21, 29, 43–45] (*P*_non−linear_ = 0.376; Fig. 3A). With per 1 unit increase in TG/HDL-C, arterial stiffness risk increased by 26% (OR 1.26, 95% CI 1.14–1.39, *I*^2^ = 61.8%, *P*_heterogeneity_ = 0.002; Fig. 4). With sensitivity analysis, no individual studies altered the summary OR significantly. Publication bias was revealed through the Egger’s tests (*P* = 0.001) and funnel plots (**Supplemental Fig. 4A**). After adjusting for publication bias, the summary finding was robust (OR 1.16, 95% CI 1.05–1.28).

Additionally, TG/HDL-C was positively related to PWV (β 0.09, 95% CI 0.04–0.14, *I*^2^ = 38.4%, *P*_heterogeneity_ = 0.165; **Supplemental Fig. 4**) [14, 29, 47].

#### TyG and arterial stiffness risk

The meta-analysis included 13 studies to evaluate associations of arterial stiffness with the highest versus lowest TyG [16–18, 22, 25, 46, 49, 51, 52]. The pooled OR of the highest TyG level was 1.86 (95% CI 1.53–2.25, *I*^2^ = 73.7%, *P*_heterogeneity_ < 0.001; Fig. 2). With sensitivity analysis, the summary results were consistent with original estimates. The Egger’s tests (*P* = 0.111) and funnel plots did not reveal publication bias (**Supplemental Fig. 4B**).

Further, with 21 records included in the dose-response meta-analysis, we discovered a positive linear relationship of TyG with arterial stiffness risk [16, 17, 19, 22, 25, 29, 46, 48–52] (*P*_non−linear_ = 0.289; Fig. 3B). With per 1 unit increase of TyG, the pooled arterial stiffness risk increased by 57% (OR 1.57, 95% CI 1.36–1.82, *I*^2^ = 94.1%, *P*_heterogeneity_ < 0.001; Fig. 4). With sensitivity analysis, the size and direction of pooled results also remained similar. The Egger’s tests (*P* < 0.001) and funnel plots all detected publication bias (**Supplemental Fig. 4B**). After adjustment, the major findings were not affected substantially (OR 1.10, 95% CI 0.97–1.26).

Moreover, TyG was positively related to PWV levels with the pooled β value of 0.57 (95% CI 0.35–0.78, *I*^2^ = 97.2%, *P*_heterogeneity_ < 0.001; **Supplemental Fig. 5**) [14, 17, 22, 25, 29, 49, 51, 53].

#### Subgroup analysis

Given the restricted number of studies, subgroup analysis was only performed between TG/HDL-C and TyG and arterial stiffness in the dose-response analysis. The overall findings of subgroup analyses confirmed that the main findings were robust, though we discovered potential sources of heterogeneity (**Supplemental Table 9**). For TG/HDL-C, we found that age, study quality, and an adjusted variable (FPG) may be sources of heterogeneity (all *P* < 0.05) by univariable meta-regression. For TyG, the heterogeneity was reduced among subgroups stratified by gender, sample size, and an adjustment factor (SBP), all of which may be potential sources of heterogeneity (all *P* < 0.05).

## Discussion

In the cross-sectional analysis, both TG/HDL-C and TyG index were positively correlated with arterial stiffness and PWV levels. The validity and generalizability of the findings were confirmed by the subsequent meta-analysis, indicating TG/HDL-C and TyG could be reliable predictors of arterial stiffness.

Although the correlations of IR with arterial stiffness have been discussed for decades, the relationships of IR with arterial stiffness are still being debated. Several studies have illustrated that TG/HDL-C and TyG are related to arterial stiffness [17–19]. We observed consistent positive and linear associations between TG/HDL-C and TyG and arterial stiffness. Meanwhile, significant interaction between SBP and TyG for arterial stiffness was observed, which is consistent with a recent meta-analysis [54].

Our meta-analysis included 40 studies, along with the current study, to assess associations of TG/HDL-C and TyG with arterial stiffness. The results suggested that TG/HDL-C and TyG were positively related to arterial stiffness risk and PWV levels, in line with previous reviews [23, 55]. Moreover, this meta-analysis revealed linear associations of TG/HDL-C and TyG with arterial stiffness risk, reinforcing the validity and generalizability of our findings.

To investigate the source of heterogeneity, a series of subgroup analyses were conducted in the dose-response analysis. Despite the high heterogeneity, the findings of subgroup and sensitivity analyses generally supported our major results. For TG/HDL-C, the heterogeneity may be due to variations in age, study quality, and a confounding factor (FPG). The study of Ungvari et al. found that age-induced pathological alterations in vascular structure and function may result in the genesis of arterial stiffness [56]. Moreover, it is well-documented that diabetes, measured by FPG, plays a crucial role in increasing arterial stiffness risk [57]. Regarding TyG, the high heterogeneity might result from differences in sex, sample size, and an adjusted variable (SBP). It seems reasonable to assume that sex-related discrepancies in hormonal status and adipose tissue distribution could explain the differences between sexes [58, 59]. The difference in SBP may be explained by SBP being associated with progression of arterial stiffness [54].

Several biological mechanisms may explain relationships of TG/HDL-C and TyG with arterial stiffness and PWV levels. To date, emerging studies have demonstrated that both excessive activation of insulin receptor and vascular mineralocorticoid receptors (MR), as occurs in a state of IR, are now recognized as playing a critical role in increasing Na^+^ channel (EnNaC) activity and expression via mTOR and SGK-1-dependent mechanisms [60]. Further, current data from epidemiological and experimental studies support the notion that over-activation of the EnNaC is associated with a series of negative consequences, including decreased NO bio-availability [61, 62], endothelial cell stiffening [63, 64], impaired vasodilator function [65, 66], oxidative stress, and stimulation of an inflammatory environment [62, 64], all of which contribute to the genesis of vascular fibrosis and stiffness [55, 60, 62–64]. In addition, IR may directly lead to vascular endothelial dysfunction [67], which has been implicated in the pathogenesis of arterial stiffness [55].

The current study has some strengths. First, the study provided the most up-to-date pooled estimates, and it performed dose-response analysis. Second, the study combined cross-sectional study with dose response meta-analysis, which provided more robust evidence. Moreover, we found positive linear associations which support the finding that higher TG/HDL-C and TyG are related to increased arterial stiffness risk. Finally, this study was conducted with TG/HDL-C and TyG analyzed separately as categorical variables and continuous variables, with similar results observed, further validating the stability of our main findings.

The present study has certain limitations, however, that should be acknowledged when interpreting its results. First, although baPWV was the commonly-used measure of arterial stiffness due to its simplicity and non-invasiveness, it could be affected by stiffness of peripheral arteries, making it less effective [68]. Second, although our findings indicated a more profound relationship between the TyG index and arterial stiffness compared to TG/HDL-C, the difference was not significant. Further research is needed to explore the potential of this finding. Third, although medication may be a confounding factor, we failed to adjust for it due to a number of missing values in medication history. The relationship can be clarified in a future study. Fourth, several studies were based on populations with diabetes or hypertension, which may be another cause of potential bias, although results of subgroup analysis were robust. Eventually, there was evidence of heterogeneity or publication bias; however, the overall findings were unaffected significantly after the trim and fill adjustment. In light of the above factors, more relevant studies are needed to clarify the relationships of TG/HDL-C and TyG with arterial stiffness risk and PWV levels.

## Conclusion

Taken together, our results indicate that TG/HDL-C and TyG may be reliable predictors of arterial stiffness risk, while TyG is positively associated with PWV levels. Given the convenience of measuring TG/HDL-C and TyG in clinical settings, further research should focus on determining whether inclusion of TG/HDL-C and TyG measures can improve the efficacy of current arterial stiffness prediction tools.

**Highlights**.

1. TG/HDL-C and TyG index were positively associated with risk of arterial stiffness and PWV levels.
2. TG/HDL-C and TyG index may be convincing predictors of arterial stiffness that could be used in clinical practice.
3. This study refined and expanded upon findings of associations of TG/HDL-C and TyG index with arterial stiffness risk.

### Electronic supplementary material

Below is the link to the electronic supplementary material.

Supplementary Material 1

## Data Availability

No datasets were generated or analysed during the current study.

## Declarations

### Original publication

The manuscript has not been published and is not being considered for publication elsewhere, in whole or in part, in any language.
