# Supplementary material for: Dose-response associations of triglyceride to high-density lipoprotein cholesterol ratio and triglyceride–glucose index with arterial stiffness risk
Source: Lipids Health Dis. 2024 Apr 20;23:115. doi: 10.1186/s12944-024-02095-z (PMC11031917; doi:10.1186/s12944-024-02095-z)
Supplement: Supplementary file 1 — Supplementary Material 1 [file 12944_2024_2095_MOESM1_ESM.docx]

# Supplemental Table Legend:

Supplemental Table 1. Sensitivity analysis of TG/HDL-C and TyG index with arterial stiffness (baPWV ≥ 1400 cm/s)

Supplemental Table 2. Interaction effects of blood pressure and FPG with TG/HDL and TyG index for arterial stiffness

Supplemental Table 3. baPWV (m/s) percentiles for males and females.

Supplemental Table 4. Associations of TG/HDL-C and TyG index with vascular aging.

Supplemental Table 5. Details of the search terms

Supplemental Table 6. Characteristics of studies included in the meta-analysis.

Supplemental Table 7. Assessment of quality of cross-sectional studies (Agency for Healthcare Research and Quality scale).

Supplementa1 Table 8. Assessment of quality of cohort studies (Newcastle-Ottawa Quality Assessment Scale).

Supplementa1 Table 9. Subgroup analyses of TG/HDL-C and TyG index with risk of arterial stiffness.

# Supplementary Figure legend:

Supplemental Figure 1. Flowchart of participant selection

Supplemental Figure 2. Flowchart of study selection

Supplemental Figure 3. Funnel plots of the highest versus lowest category of A: TG/HDL-C and B: TyG index with risk of arterial stiffness.

Supplemental Figure 4. Funnel plots per 1 unit increment in A: TG/HDL-C and B: TyG index with risk of arterial stiffness.

Supplemental Figure 5. Forest plot of pooled association between TG/HDL-C and TyG index (per 1 unit increment) and baPWV levels (m/s).

**Supplemental Table 1.** Sensitivity analysis of TG/HDL-C and TyG index with arterial stiffness (baPWV ≥ 1400 cm/s)

|  | **Range** | **Model 1** | **Model 2** | **Model 3** |
| --- | --- | --- | --- | --- |
| **TG/HDL-C** |  |  |  |  |
| Q1 | <0.73 | Ref | Ref | Ref |
| Q2 | 0.73-1.11 | 1.14 (0.85-1.52) | 1.22 (0.83-1.49) | 1.06 (0.76-1.49) |
| Q3 | 1.11-1.83 | 1.62 (1.20-2.18) | 1.61 (1.19-2.18) | 1.64 (1.15-2.33) |
| Q4 | ≥1.83 | 1.66 (1.24-2.23) | 1.56 (1.15-2.10) | 1.50 (1.05-2.15) |
| *P* for trend |  | <0.001 | <0.001 | 0.005 |
| Per 1 unit increase |  | 1.12 (1.03-1.21) | 1.10 (1.02-1.19) | 1.11 (1.01-1.22) |
| **TyG index** |  |  |  |  |
| Q1 | <8.45 | Ref | Ref | Ref |
| Q2 | 8.45-8.81 | 1.33 (1.00-1.78) | 1.33 (0.99-1.78) | 1.35 (0.96-1.89) |
| Q3 | 8.81-9.23 | 2.18 (1.61-2.94) | 2.12 (1.57-2.88) | 2.03 (1.42-2.92) |
| Q4 | ≥9.23 | 1.86 (1.61-2.94) | 1.76 (1.30-2.38) | 1.69 (1.17-2.45) |
| *P* for trend |  | <0.001 | <0.001 | 0.001 |
| Per 1 unit increase |  | 1.67 (1.39-2.02) | 1.61 (1.34-1.95) | 1.57 (1.25-1.98) |

Data are presented as odds ratio (ORs) and 95% confidence intervals (95% CIs).

Model 1: adjusted for age and gender.

Model 2: adjusted for age, gender, marital status, education, smoking, alcohol drinking, and physical activity.

Model 3: adjusted for age, gender, marital status, education, smoking, alcohol drinking, physical activity, body mass index, waist circumference, systolic blood pressure, diastolic blood pressure, and total cholesterol.

**Supplemental Table 2.** Interaction effects of blood pressure and FPG with TG/HDL and TyG index for arterial stiffness

| Variables | *P* _interaction_ for TG/HDL-C | *P* _interaction_ for TyG index |
| --- | --- | --- |
| SBP, mmHg | 0.076 | 0.032 |
| DBP, mmHg | 0.345 | 0.399 |
| FPG, mmol/L | 0.197 | 0.853 |

Note: interaction effects were based on model adjusted age, marital status, education, smoking, alcohol drinking, physical activity, body mass index, waist circumference, SBP, DBP, total cholesterol, and FPG.

Abbreviations: SBP, systolic blood pressure; DBP, diastolic blood pressure; FPG, fasting plasma glucose.

**Supplemental Table 3.** baPWV (m/s) percentiles for males and females.

| Age, years | N | baPWV (m/s) | | | | | | | | |
| --- | --- | --- | --- | --- | --- | --- | --- | --- | --- | --- |
|  |  | 1st | 5th | 10th | 25th | 50th | 75th | 90th | 95th | 99th |
| Males | 716 |  |  |  |  |  |  |  |  |  |
| 18-44 | 45 | 10.83 | 11.44 | 12.07 | 12.69 | 13.04 | 14.21 | 15.12 | 16.00 | 17.54 |
| 45-60 | 246 | 10.15 | 11.35 | 11.90 | 12.78 | 13.99 | 15.47 | 17.31 | 18.96 | 20.98 |
| > 60 | 425 | 10.93 | 12.28 | 13.00 | 14.28 | 16.03 | 18.62 | 20.83 | 22.27 | 25.08 |
| Females | 1263 |  |  |  |  |  |  |  |  |  |
| 18-44 | 85 | 9.83 | 10.06 | 10.49 | 11.36 | 12.21 | 13.28 | 14.59 | 14.94 | 17.93 |
| 45-60 | 504 | 10.10 | 10.83 | 11.39 | 12.49 | 13.98 | 15.59 | 16.99 | 18.15 | 20.17 |
| > 60 | 674 | 11.47 | 12.41 | 13.35 | 14.87 | 16.90 | 19.08 | 21.27 | 22.55 | 25.62 |

Abbreviations: baPWV, brachial-ankle pulse wave velocity.

**Supplemental Table 4.** Associations of TG/HDL-C and TyG index with vascular aging.

|  | **Range** | **Model 1** | **Model 2** | **Model 3** |
| --- | --- | --- | --- | --- |
| **TG/HDL-C** |  |  |  |  |
| Q1 | <0.73 | Ref | Ref | Ref |
| Q2 | 0.73-1.11 | 1.34 (0.74-2.42) | 1.62 (1.08-2.43) | 2.52 (0.93-6.81) |
| Q3 | 1.11-1.83 | 1.64 (0.93-2.91) | 2.85 (1.78-4.58) | 5.16 (1.80-14.84) |
| Q4 | ≥1.83 | 1.87 (1.05-3.32) | 3.26 (1.97-5.40) | 8.08 (2.75-23.70) |
| *P* for trend |  | 0.026 | < 0.001 | < 0.001 |
| Per 1 unit increase |  | 1.02 (0.90-1.16) | 1.47 (1.20-1.79) | 1.87 (1.30-2.68) |
| **TyG index** |  |  |  |  |
| Q1 | <8.45 | Ref | Ref | Ref |
| Q2 | 8.45-8.81 | 1.22 (0.66-2.24) | 1.94 (1.27-2.94) | 3.71 (1.27-10.81) |
| Q3 | 8.81-9.23 | 1.63 (0.91-2.91) | 2.89 (1.78-4.68) | 6.28 (1.98-19.94) |
| Q4 | ≥9.23 | 2.04 (1.14-3.68) | 2.94 (1.75-4.93) | 7.85 (2.52-24.51) |
| *P* for trend |  | 0.009 | < 0.001 | < 0.001 |
| Per 1 unit increase |  | 1.61 (1.17-2.20) | 2.35 (1.65-3.35) | 4.36 (2.21-8.62) |

Data are presented as odds ratio (ORs) and 95% confidence intervals (95% CIs). And in the three models, the adjusted variables were age, gender, marital status, education, smoking, alcohol drinking, physical activity, body mass index, waist circumference, systolic blood pressure, diastolic blood pressure, and total cholesterol.

Model 1: HVA and NVA were coded as 0, and EVA was coded 1.

Model 2: HVA was coded 0, and HVA and EVA were coded 1.

Model 3: HVA was coded 0, and EVA was coded 1.

**Supplemental Table 5.** Details of the search terms

| **Search terms for PubMed (1345 articles)** |
| --- |
| #1 "insulin resistance"[MeSH Terms] OR "insulin resistance"[Title/Abstract] OR "insulin sensitivity"[Title/Abstract] OR "homeostasis model assessment"[Title/Abstract] OR "HOMA"[Title/Abstract] OR "HOMA-IR"[Title/Abstract] |
| #2 "TyG"[Title/Abstract] OR "tyg index"[Title/Abstract] OR "triglyceride glucose index"[Title/Abstract] OR ("triglyceride"[Title/Abstract] AND "glucose index"[Title/Abstract]) OR "triglyceride glucose index"[Title/Abstract] OR (("triglycerides"[MeSH Terms] OR "triglycerides"[All Fields] OR "triacylglycerol"[All Fields] OR "triacylglycerols"[All Fields]) AND "glucose index"[Title/Abstract]) |
| #3 "triglyceride high density lipoprotein cholesterol ratio"[Title/Abstract] OR "triglyceride to high density lipoprotein cholesterol ratio"[Title/Abstract] OR " TG/HDL-C "[Title/Abstract] OR " TG/HDL cholesterol"[Title/Abstract] |
| #4 #1 OR #2 OR #3 |
| #5 "vascular stiffness"[MeSH Terms] OR "vascular stiffness"[Title/Abstract] OR "arterial stiffness"[Title/Abstract] OR "aortic stiffness"[Title/Abstract] OR "arterial compliance"[Title/Abstract] OR "arterial elasticity"[Title/Abstract] OR "artery compliance"[Title/Abstract] OR "pulse wave analysis"[MeSH Terms] OR "pulse wave analysis"[Title/Abstract] OR "pulse wave velocity"[Title/Abstract] OR "PWV"[Title/Abstract] |
| #4 AND #5 |
| **Search terms for Embase (2467 articles)** |
| #1 insulin resistance/ OR insulin sensitivity/ OR homeostasis model assessment/ OR insulin resistance.mp. OR insulin sensitivity.mp. OR homeostasis model assessment.mp. OR HOMA.mp. OR HOMA-IR.mp. |
| #2 TyG.mp. OR TyG index.mp. OR triglyceride glucose index.mp. OR triglyceride-glucose index.mp. OR triacylglycerol glucose index.mp. |
| #3 triglyceride high density lipoprotein cholesterol ratio.mp. OR triglyceride to high density lipoprotein cholesterol ratio.mp. OR TG HDL-C.mp. OR TG HDL cholesterol.mp. |
| #4 #1 OR #2 OR #3 |
| #5 arterial stiffness/ OR artery compliance/ OR pulse wave velocity/ OR vascular stiffness.mp. OR arterial stiffness.mp. OR aortic stiffness.mp. OR arterial compliance.mp. OR arterial elasticity.mp. OR artery compliance.mp. OR pulse wave analysis.mp. OR pulse wave velocity.mp. OR PWV.mp. |
| #4 AND #5 |
| **Search terms for Web of Science (2529 articles)** |
| #1 TS= ("insulin resistance" OR "insulin sensitivity" OR "homeostasis model assessment" OR "HOMA" OR "HOMA-IR") |
| #2 TS= ("TyG" OR "TyG index" OR "triglyceride glucose index" OR "triglyceride and glucose index" OR "triglyceride glucose index" OR "triacylglycerol glucose index") |
| #3 TS= ("triglyceride high density lipoprotein cholesterol ratio" OR "triglyceride to high density lipoprotein cholesterol ratio" OR "TG/HDL-C" OR "TG/HDL cholesterol") |
| #4 #1 OR #2 OR #3 |
| #5 TS= ("vascular stiffness" OR "arterial stiffness" OR "aortic stiffness" OR "arterial compliance" OR "arterial elasticity" OR "artery compliance" OR "pulse wave analysis" OR "pulse wave velocity" OR "PWV") |
| #4 AND #5 |

**Supplemental Table 6.** Characteristics of studies included in the meta-analysis.

| Study | Country | Study design | Sample Size | Follow-up (years) | Age, years (mean or range) | Cases | Exposure | Adjustment |
| --- | --- | --- | --- | --- | --- | --- | --- | --- |
| Zhao et al. (2014) ^[20]^ | China | Cross-sectional | 1,133 (430 males) | NA | 50-90 | NA | TG/HDL-C | Age, sex |
| Wen et al. (2015) ^[43]^ | China | Cross-sectional | 1,498 (926 males) | NA | NA | NA | TG/HDL-C | Age, body mass index, systolic blood pressure, diastolic blood pressure, low-density lipoprotein cholesterol, fasting plasma glucose, uric acid, and estimated glomerular filtration rate |
| Wen et al. (2017) ^[44]^ | China | Cross-sectional | 1,015 males | NA | 18-44 | NA | TG/HDL-C | Age, systolic and diastolic blood pressure, alanine aminotransferase, aminotransferase, γ-glutamyl transpeptidase, and fasting glucose. |
| Li et al. (2018) ^[45]^ | China | Cohort | 816 (476 males) | 5 | 39.1 | 144 | TG/HDL-C | Age, gender, physical activity, smoking, drinking, body mass index, systolic blood pressure, mean arterial pressure, fasting blood glucose, uric acid, baseline pulse wave velocity, tumor necrosis factor α, high-sensitivity C reactive protein, and Interleukin-6. |
| Lee et al. (2018) ^[46]^ | Korea | Cross-sectional | 3,587 (2,061 males) | NA | 54-64 | NA | TyG index | Age, systolic blood pressure, body mass index, low-density lipoprotein cholesterol, low-density lipoprotein cholesterol, diabetes mellitus, and menopause (women). |
| Chen et al. (2018) ^[47]^ | Japan | Cross-sectional | 912 (592 males) | NA | 51.1 | NA | TG/HDL-C | Age, sex, body mass index, systolic blood pressure, diastolic blood pressure, aspartate aminotransferase, alanine  aminotransferase, γ-glutamyl transpeptidase, uric acid, fasting glucose, total cholesterol, low density lipoprotein, estimated glomerular filtration rate, smoking and exercise status, fatty liver, alcohol consumption, ankle-brachial index. |
| Chung et al. (2019) ^[15]^ | Korea | Cross-sectional | 434 females | NA | ≥46 | NA | TG/HDL-C | Age, body mass index, smoking status, regular exercise, mean arterial pressure, fasting plasma glucose, total cholesterol level, hypertension, log‐transformed C‐reactive protein, and the use of antihypertensive and lipid lowering drugs. |
| Zhao et al. (2019) ^[18]^ | US | Cross-sectional | 2,830 (1,571 males) | NA | ≥65 | NA | TyG index | Age, sex, body mass index, waist circumference, smoking habit, hypertension, family history of premature cardiovascular disease, diabetes, high-density lipoprotein cholesterol, low-density lipoprotein cholesterol, insulin therapy and statin therapy. |
| Poon et al. (2020) ^[16]^ | US | Cohort | 2,571 (957 males) | NA | 67-90 | 640 | TG/HDL-C | Age, gender (except for gender-specific estimates), and race/study-site. |
|  |  |  |  |  |  |  | TyG index |  |
| Nakagomi et al. (2020) ^[19]^ | Japan | Cross-sectional | 2,818 (1,720 males) | NA | 25-55 | NA | TG/HDL-C | Age, body mass index, systolic blood pressure, HbA1c, fasting blood glucose, low density lipoprotein cholesterol, high density lipoprotein cholesterol, uric acid, smoking status (never, past, current), and alcohol drinking (never, past, current). |
|  |  |  |  |  |  |  | TyG index |  |
| Li et al. (2020) ^[17]^ | China | Cross-sectional | 4,718 (2,346 males) | NA | ≥18 | NA | TyG index | Age, sex, education, body mass index, waist circumference, physical activity, current smoking, current drinking, systolic blood pressure, diastolic blood pressure, serum uric acid, serum homocysteine, high-density lipoprotein cholesterol, low-density lipoprotein cholesterol, estimated glomerular filtration rate, self-reported diabetes, antihypertensive drugs, antiplatelet drugs. |
| Guo et al. (2021) ^[48]^ | China | Cross-sectional | 13,706 (7,633 males) | NA | 49.4 | NA | TyG index | Age, smoking, body mass index, pulse pressure, HbA1c, total cholesterol, low-density lipoprotein cholesterol, high-density lipoprotein cholesterol, uric acid, and antihypertensive medication status. |
| Wang et al. (2021) ^[49]^ | China | Cross-sectional | 3,185 (1,954 males) | NA | 54.6 | NA | TyG index | Age, sex, body mass index, waist circumference, systolic blood pressure, low-density lipoprotein cholesterol, high-density lipoprotein cholesterol, white blood cell counts, smoking status, drinking status, lipid lowering agents, antihypertensive agents, insulin therapy, non-insulin hypoglycemic agents. |
| Kılıç et al. (2021) ^[21]^ | Turkey | Cross-sectional | 1,582 (504 males) | NA | 52.8 | NA | TG/HDL-C | Age, sex, body mass index, diabetes mellitus, current smoking, triglyceride, non-high-density lipoprotein cholesterol. |
| Wu et al. (2021) ^[14]^ | China | Cohort | 1,895 (1,477 males) | 4.71 | 61.9 | NA | TG/HDL-C | Age, sex, body mass index, smoking status, drinking status, physical activity, diabetes, dyslipidaemia, brachial-ankle pulse wave velocity at baseline, mean arterial pressure at baseline and follow-up, fasting blood glucose, triglyceride, postprandial blood glucose, low-density lipoprotein cholesterol, estimated glomerular filtration rate, uric acid, homocysteine, and use of antidiabetic, lipid-lowering, or antihypertensive medications at baseline and follow-up. |
|  |  |  |  |  |  |  | TyG index |  |
| Wu et al. (2021) ^[25]^ | China | Cohort | 6,028 (3,181 males) | 26,839* | 47 | 680 | TyG index | Age, sex, smoking, alcohol drinking, physical activity, mean arterial pressure, diabetes, high-sensitivity C-reactive protein, and body mass index. |
| Su et al. (2021) ^[22]^ | China | Cross-sectional | 2,035 (1,229 males) | NA | 61-95 | NA | TyG index | Age, sex, body mass index, waist circumference, systolic blood pressure, diastolic blood pressure, total cholesterol, high-density lipoprotein cholesterol, low-density lipoprotein cholesterol, uric acid, estimated glomerular filtration rate, smoking status, drinking status, coronary heart disease, hypertension, diabetes mellitus, anti-platelet agents, anti-hypertensive agents, hypoglycemic therapy, and lipid-lowering therapy. |
| Pan et al. (2021) ^[50]^ | China | Cross-sectional | 4,721 (2,529 males) | 5 | 40-69 | NA | TyG index | Age, sex, body mass index, and smoking status. |
| Gómez-Sánchez et al. (2021) ^[29]^ | Spain | Cross-sectional | 501 (251 males) | NA | 35-75 | NA | TG/HDL-C | Age, sex, antihypertensive, lipid-lowering and hypoglycemic drugs |
|  |  |  |  |  |  |  | TyG index |  |
| Ji et al. (2022) ^[51]^ | China | Cohort | 6,015 (2,064 males) | 7 | 62.4 | 1028 | TyG index | Age, sex, smoking and drinking habits, BMI, SBP, DBP, HDL-C, LDL-C, eGFR, self-reported CHD and stroke, antihypertensive drugs, hypoglycemic drugs, lipid-lowering drugs. |
| Yang et al. (2022) ^[52]^ | Japan | Cross-sectional | 912 (592 males) | NA | 51.1 | NA | TyG index | Age, sex, BMI, SBP, DBP, HDL-C, fatty liver, eGFR, |
| Muhammad et al. (2023) ^[53]^ | Swedish | Cohort | 2,697 (1,028 males) | 16.9 | 56 |  |  | Sex, MAP, heart rate and age at follow-up, baseline age, smoking habits, systolic blood pressure, waist circumference, diabetes, use of anti-hypertensive medication, and use of lipid-lowering medication, HDL, LDL, CRP. |
| Zhang et al. (2024) | China | Cross-sectional | 1,979 (716 males) | NA | 62 | 414 | TG/HDL-C | Age, gender, marital status, education, smoking, alcohol drinking, physical activity, BMI, WC, SBP, DBP, TC, lipid-lowering therapy. |
|  |  |  |  |  |  |  | TyG index |  |

*: person-years.

Note: US, United States; NA, Not applicable; TG/HDL-C, Triglyceride to high-density lipoprotein cholesterol ratio; TyG index, Triglyceride-glucose index.

**Supplemental Table 7.** Assessment of quality of cross-sectional studies (Agency for Healthcare Research and Quality scale).

| First author (year) | Term 1 | Term 2 | Term 3 | Term 4 | Term 5 | Term 6 | Term 7 | Term 8 | Term 9 | Term 10 | Term 11 | Total |
| --- | --- | --- | --- | --- | --- | --- | --- | --- | --- | --- | --- | --- |
| Zhao et al. (2014) ^[20]^ | 1 | 1 | 1 | 1 | 0 | 1 | 1 | 0 | 1 | 0 | 0 | 7 |
| Wen et al. (2015) ^[42]^ | 1 | 1 | 1 | 1 | 0 | 1 | 1 | 0 | 1 | 0 | 0 | 7 |
| Wen et al. (2017) ^[43]^ | 1 | 1 | 1 | 1 | 0 | 1 | 1 | 0 | 1 | 0 | 0 | 7 |
| Lee et al. (2018) ^[44]^ | 1 | 1 | 1 | 1 | 0 | 1 | 1 | 0 | 1 | 0 | 0 | 7 |
| Chen et al. (2018) ^[45]^ | 1 | 1 | 1 | 1 | 0 | 1 | 1 | 1 | 1 | 0 | 0 | 8 |
| Chung et al. (2019) ^[15]^ | 1 | 1 | 0 | 1 | 0 | 1 | 1 | 0 | 1 | 0 | 0 | 6 |
| Zhao et al. (2019) ^[18]^ | 1 | 1 | 1 | 1 | 0 | 1 | 1 | 1 | 1 | 1 | 0 | 9 |
| Poon et al. (2020) ^[16]^ | 1 | 1 | 1 | 1 | 0 | 1 | 1 | 1 | 1 | 1 | 0 | 9 |
| Nakagomi et al. (2020) ^[19]^ | 1 | 1 | 1 | 1 | 0 | 1 | 1 | 0 | 1 | 0 | 0 | 7 |
| Li et al. (2020) ^[17]^ | 1 | 1 | 1 | 1 | 0 | 1 | 1 | 0 | 1 | 0 | 0 | 7 |
| Guo et al. (2021) ^[47]^ | 1 | 1 | 1 | 1 | 0 | 1 | 1 | 1 | 0 | 1 | 0 | 8 |
| Wang et al. (2021) ^[48]^ | 1 | 1 | 1 | 1 | 0 | 1 | 1 | 1 | 1 | 1 | 0 | 9 |
| KILIÇ et al. (2021) ^[21]^ | 1 | 1 | 1 | 1 | 0 | 1 | 1 | 0 | 1 | 1 | 0 | 8 |
| Su et al. (2021) ^[22]^ | 1 | 1 | 1 | 1 | 0 | 1 | 1 | 1 | 1 | 1 | 0 | 9 |
| Pan et al. (2021) ^[49]^ | 1 | 1 | 0 | 1 | 0 | 1 | 1 | 0 | 1 | 0 | 0 | 6 |
| Gómez-Sánchez et al. (2021) ^[50]^ | 1 | 1 | 1 | 1 | 0 | 1 | 1 | 1 | 0 | 1 | 0 | 8 |
| Yang et al. (2022) ^[52]^ | 1 | 1 | 1 | 1 | 0 | 1 | 1 | 1 | 1 | 1 | 0 | 9 |
| Zhang et al. (2023) | 1 | 1 | 1 | 1 | 0 | 1 | 1 | 1 | 1 | 1 | 0 | 9 |

Term1. Define the source of information (survey, record review).

Term2. List inclusion and exclusion criteria for exposed and unexposed subjects (cases and controls) or refer to previous publications.

Term3. Indicate time period used for identifying patients.

Term4. Indicate whether or not subjects were consecutive if not population-based.

Term5. Indicate if evaluators of subject components of study were masked to other aspects of the status of the participants.

Term6. Describe any assessments undertaken for quality assurance purposes (such as test/retest of primary outcome measurements).

Term7. Explain any patient exclusion from analysis.

Term8. Describe how confounding was assessed and/or controlled.

Term9. If applicable, explain how missing data were handled in the analysis.

Term10. Summarize patient response rates and completeness of data collection.

Term11. Clarify what follow-up, if any, was expected and the percentage of patients for which incomplete data or follow-up was obtained.

**Supplementa1 Table 8.** Assessment of quality of cohort studies (Newcastle-Ottawa Quality Assessment Scale).

| First author (year) | a | b | c | d | e | f | g | h | i | Total |
| --- | --- | --- | --- | --- | --- | --- | --- | --- | --- | --- |
| Li et al. (2018) ^[44]^ | 1 | 1 | 1 | 1 | 1 | 1 | 1 | 1 | 0 | 8 |
| Wu et al. (2021) ^[14]^ | 1 | 1 | 1 | 1 | 1 | 0 | 1 | 1 | 1 | 8 |
| Wu et al. (2021) ^[25]^ | 1 | 1 | 1 | 1 | 1 | 0 | 1 | 1 | 1 | 8 |
| Ji et al. (2021) ^[51]^ | 1 | 1 | 1 | 1 | 1 | 1 | 1 | 1 | 1 | 9 |
| Muhammad et al. (2023) ^[53]^ | 1 | 1 | 1 | 1 | 1 | 1 | 1 | 1 | 1 | 9 |

a. Representativeness of the exposed cohort;

b. Selection of the non-exposed cohort;

c. Ascertainment of exposure;

d. Demonstration that outcome of interest was not present at start of study;

e. Comparability of cohorts on the basis of the design or analysis (adjusted for age);

f. Comparability of cohorts on the basis of the design or analysis (adjusted for systolic blood pressure);

g. Assessment of outcome;

h. Was follow-up long enough for outcomes to occur;

i. Adequacy of follow-up of cohort.

**Supplementa1 Table 9.** Subgroup analyses of TG/HDL-C and TyG index with risk of arterial stiffness.

| Subgroup | TG/HDL-C (per 1 unit increment) | | | | |  | TyG index (per 1 unit increment) | | | | |
| --- | --- | --- | --- | --- | --- | --- | --- | --- | --- | --- | --- |
|  | N | OR (95% CI) | *I*^2^ (%) | *P*^1^ | *P*^2^ |  | N | OR (95% CI) | *I*^2^ (%) | *P*^1^ | *P*^2^ |
| All studies | 12 | 1.26 (1.14-1.39) | 61.7 | 0.003 |  |  | 21 | 1.58 (1.36-1.82) | 94.1 | <0.001 |  |
| Age (years) |  |  |  |  | 0.011 |  |  |  |  |  | 0.548 |
| ≤55 | 7 | 1.51 (1.25-1.83) | 47.1 | 0.078 |  |  | 11 | 1.62 (1.40-1.87) | 72.6 | <0.001 |  |
| >55 | 5 | 1.14 (1.08-1.20) | 0.0 | 0.495 |  |  | 10 | 1.53 (1.24-1.89) | 94.4 | <0.001 |  |
| Sex |  |  |  |  | 0.388 |  |  |  |  |  | 0.039 |
| Men | 4 | 1.37 (1.08-1.73) | 74.7 | 0.008 |  |  | 8 | 1.71 (1.48-1.99) | 60.0 | 0.015 |  |
| Women | 3 | 2.43 (0.88-6.70) | 81.7 | 0.004 |  |  | 8 | 1.73 (1.55-1.92) | 5.7 | 0.386 |  |
| Both | 5 | 1.18 (1.07-1.31) | 30.1 | 0.221 |  |  | 5 | 1.32 (1.08-1.59) | 94.5 | <0.001 |  |
| Region |  |  |  |  | 0.153 |  |  |  |  |  | 0.096 |
| Asia | 9 | 1.44 (1.20-1.74) | 66.4 | 0.002 |  |  | 18 | 1.61 (1.46-1.78) | 70.5 | <0.001 |  |
| Europe/US | 3 | 1.14 (1.07-1.21) | 0.0 | 0.464 |  |  | 3 | 1.33 (0.90-1.97) | 88.3 | <0.001 |  |
| Sample size |  |  |  |  | 0.862 |  |  |  |  |  | 0.023 |
| ≤1,000 | 5 | 1.27 (1.07-1.50) | 73.1 | 0.005 |  |  | 5 | 1.24 (0.96-1.61) | 63.5 | 0.027 |  |
| >1,000 | 7 | 1.28 (1.11-1.48) | 56.8 | 0.031 |  |  | 16 | 1.64 (1.48-1.82) | 73.4 | <0.001 |  |
| Study design |  |  |  |  | 0.685 |  |  |  |  |  | 0.222 |
| Cross-sectional study | 11 | 1.25 (1.12-1.39) | 61.8 | 0.003 |  |  | 19 | 1.61 (1.35-1.92) | 94.2 | <0.001 |  |
| Cohort study | 1 | 1.36 (1.14-1.62) | - | - |  |  | 2 | 1.31 (1.12-1.52) | 65.1 | 0.091 |  |
| Study quality |  |  |  |  | 0.010 |  |  |  |  |  | 0.051 |
| Medium | 6 | 1.73 (1.34-2.24) | 41.2 | 0.131 |  |  | 7 | 1.81 (1.50-2.17) | 73.2 | 0.001 |  |
| High | 6 | 1.15 (1.09-1.22) | 13.2 | 0.330 |  |  | 14 | 1.46 (1.24-1.72) | 93.0 | <0.001 |  |
| PWV assessment site |  |  |  |  | 0.128 |  |  |  |  |  | 0.089 |
| baPWV | 7 | 1.60 (1.23-2.08) | 72.7 | 0.001 |  |  | 17 | 1.63 (1.46-1.82) | 71.9 | <0.001 |  |
| cfPWV | 4 | 1.17 (1.08-1.26) | 40.3 | 0.170 |  |  | 4 | 1.35 (1.01-1.79) | 91.8 | <0.001 |  |
| aPWV | 1 | 0.97 (0.55-1.70) | - | - |  |  | 0 | - | - | - |  |
| Adjusted variables |  |  |  |  |  |  |  |  |  |  |  |
| BMI |  |  |  |  | 0.440 |  |  |  |  |  | 0.096 |
| Yes | 7 | 1.38 (1.14-1.67) | 68.3 | 0.004 |  |  | 18 | 1.61 (1.46-1.78) | 70.5 | <0.001 |  |
| No | 5 | 1.18 (1.06-1.31) | 52.0 | 0.080 |  |  | 3 | 1.33 (0.90-1.97) | 88.3 | <0.001 |  |
| Smoking |  |  |  |  | 0.911 |  |  |  |  |  | 0.388 |
| Yes | 5 | 1.30 (1.06-1.61) | 70.2 | 0.009 |  |  | 14 | 1.60 (1.44-1.78) | 72.5 | <0.001 |  |
| No | 7 | 1.25 (1.10-1.41) | 60.3 | 0.019 |  |  | 7 | 1.46 (1.07-2.00) | 91.5 | <0.001 |  |
| Drinking |  |  |  |  | 0.730 |  |  |  |  |  | 0.452 |
| Yes | 4 | 1.35 (1.08-1.70) | 76.7 | 0.005 |  |  | 11 | 1.63 (1.40-1.89) | 74.0 | <0.001 |  |
| No | 8 | 1.23 (1.09-1.38) | 55.0 | 0.030 |  |  | 9 | 1.50 (1.20-1.87) | 95.2 | <0.001 |  |
| FPG |  |  |  |  | 0.006 |  |  |  |  |  | 0.417 |
| Yes | 6 | 1.57 (1.29-1.90) | 46.2 | 0.098 |  |  | 2 | 1.81 (1.29-2.55) | 22.4 | 0.256 |  |
| No | 6 | 1.13 (1.08-1.19) | 0.0 | 0.595 |  |  | 19 | 1.55 (1.33-1.80) | 94.5 | <0.001 |  |
| SBP |  |  |  |  | 0.129 |  |  |  |  |  | 0.025 |
| Yes | 7 | 1.47 (1.21-1.79) | 72.2 | 0.001 |  |  | 12 | 1.76 (1.55-1.98) | 47.6 | 0.034 |  |
| No | 5 | 1.14 (1.07-1.21) | 0.0 | 0.461 |  |  | 9 | 1.41 (1.18-1.69) | 94.8 | <0.001 |  |

*P*^1^: *P* value for heterogeneity within each subgroup.

*P*^2^: *P* value for heterogeneity between subgroups with meta-regression analysis.

Note: OR, Odds ratio; CI, confidence interval; TG/HDL-C, Triglyceride to high-density lipoprotein cholesterol ratio; TyG index, Triglyceride-glucose index; PWV, pulse wave velocity; baPWV, brachial-ankle PWV; cfPWV, carotid-femoral PWV; aPWV, aortic PWV; BMI, body mass index; FPG, fasting plasma glucose; SBP, systolic blood pressure; US, United State.


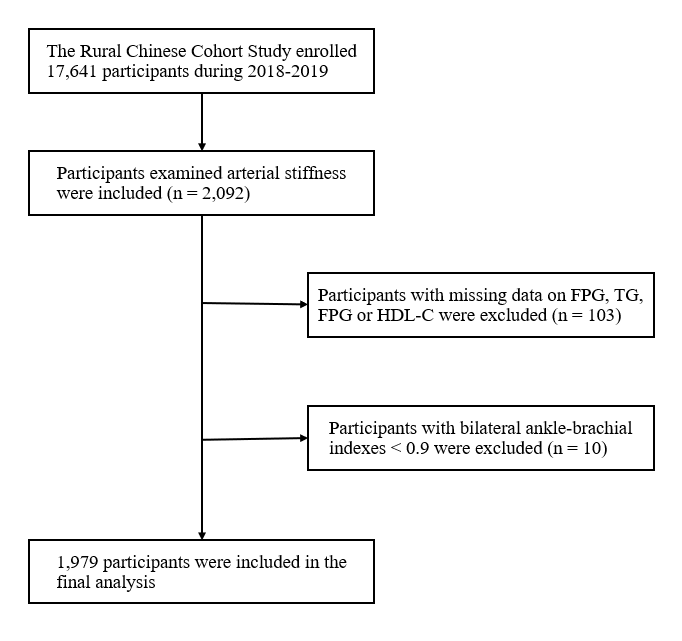


**Supplemental Figure 1**. Flowchart of participant selection


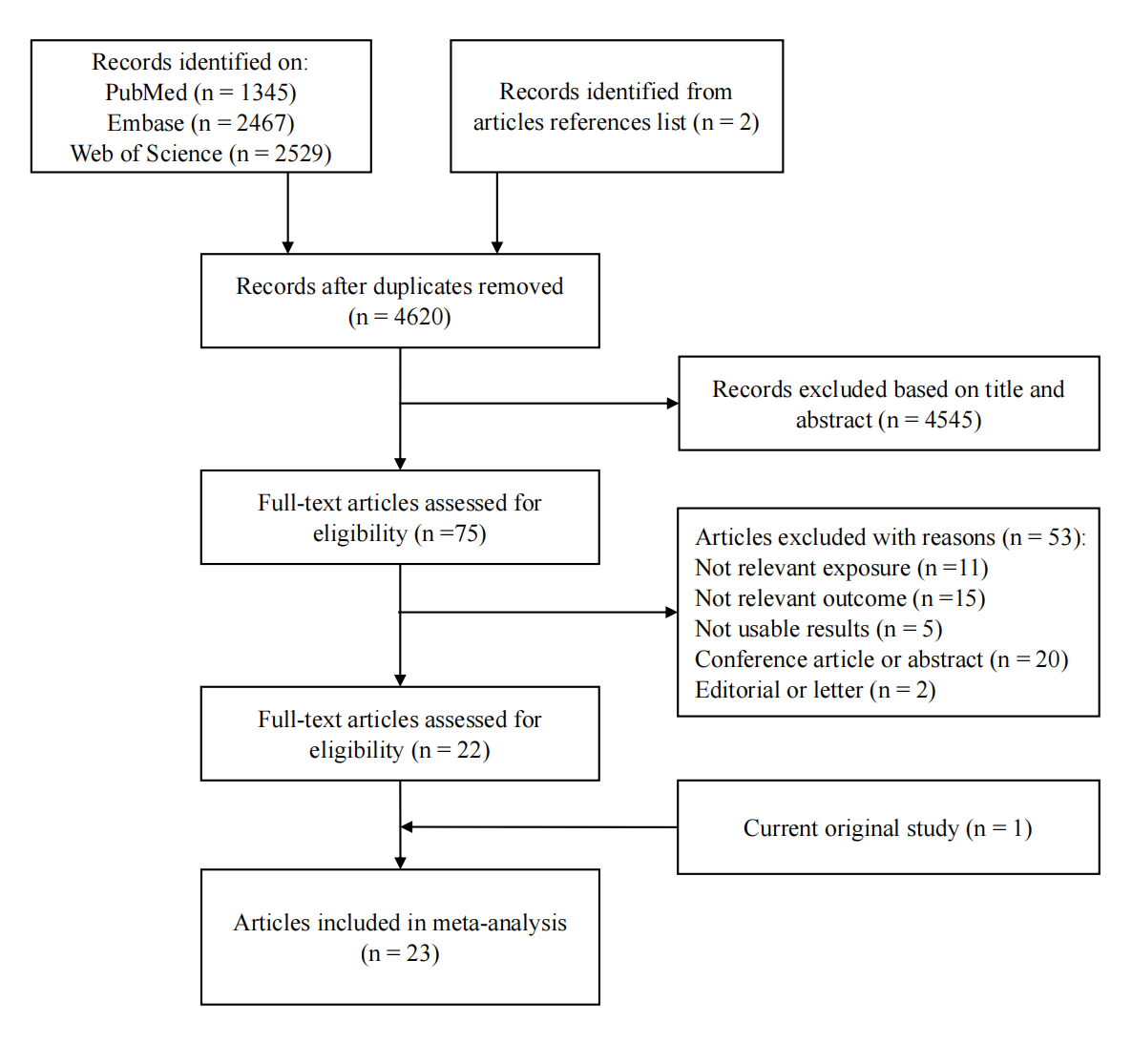


**Supplemental Figure 2**. Flowchart of study selection


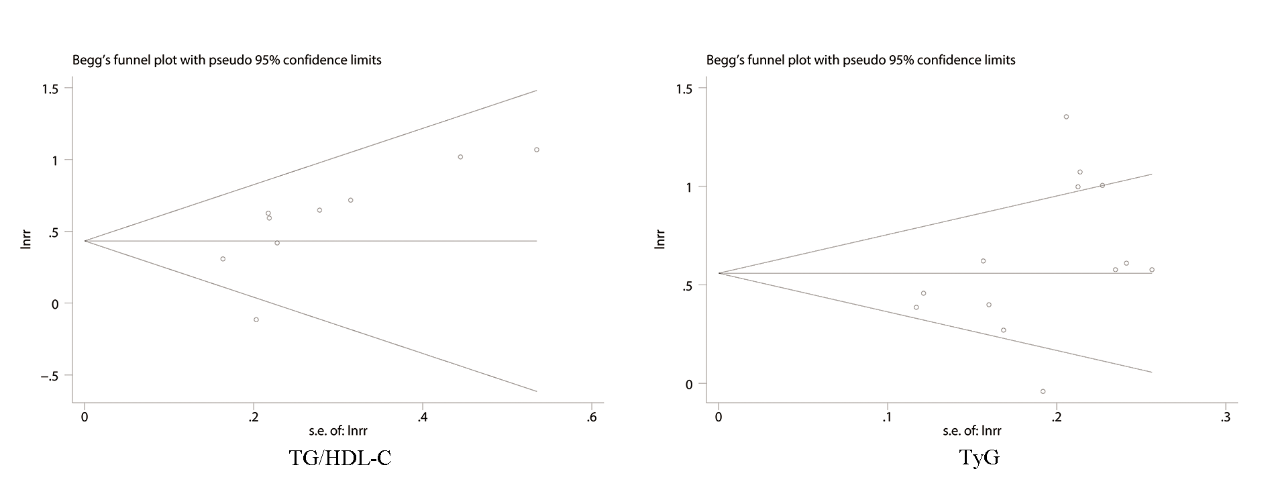


**Supplemental Figure 3.** Funnel plots of the highest versus lowest category of A: TG/HDL-C and B: TyG index with risk of arterial stiffness.


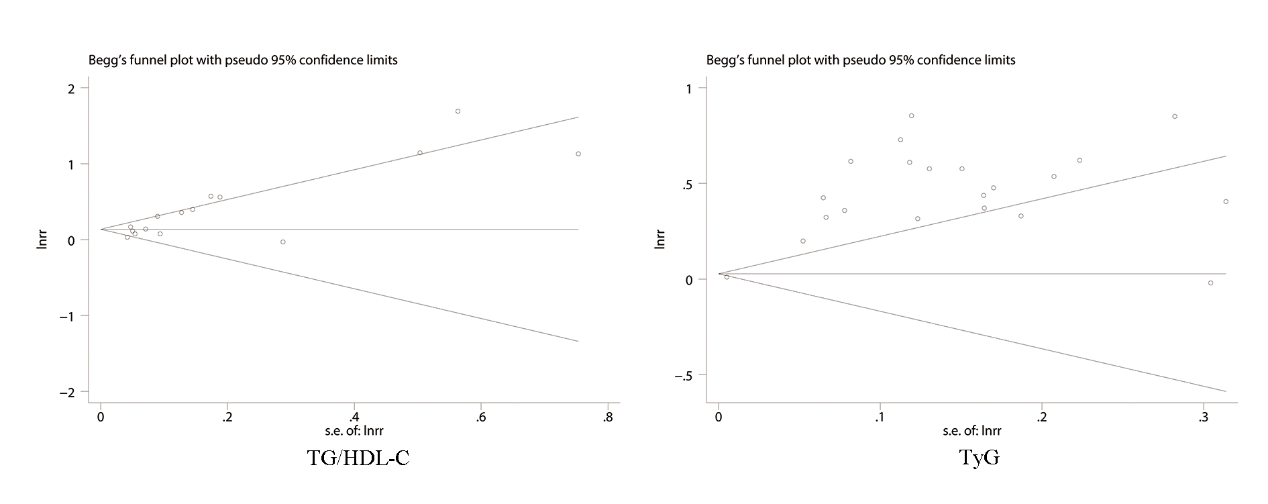


**Supplemental Figure 4.** Funnel plots per 1 unit increment in A: TG/HDL-C and B: TyG index with risk of arterial stiffness.


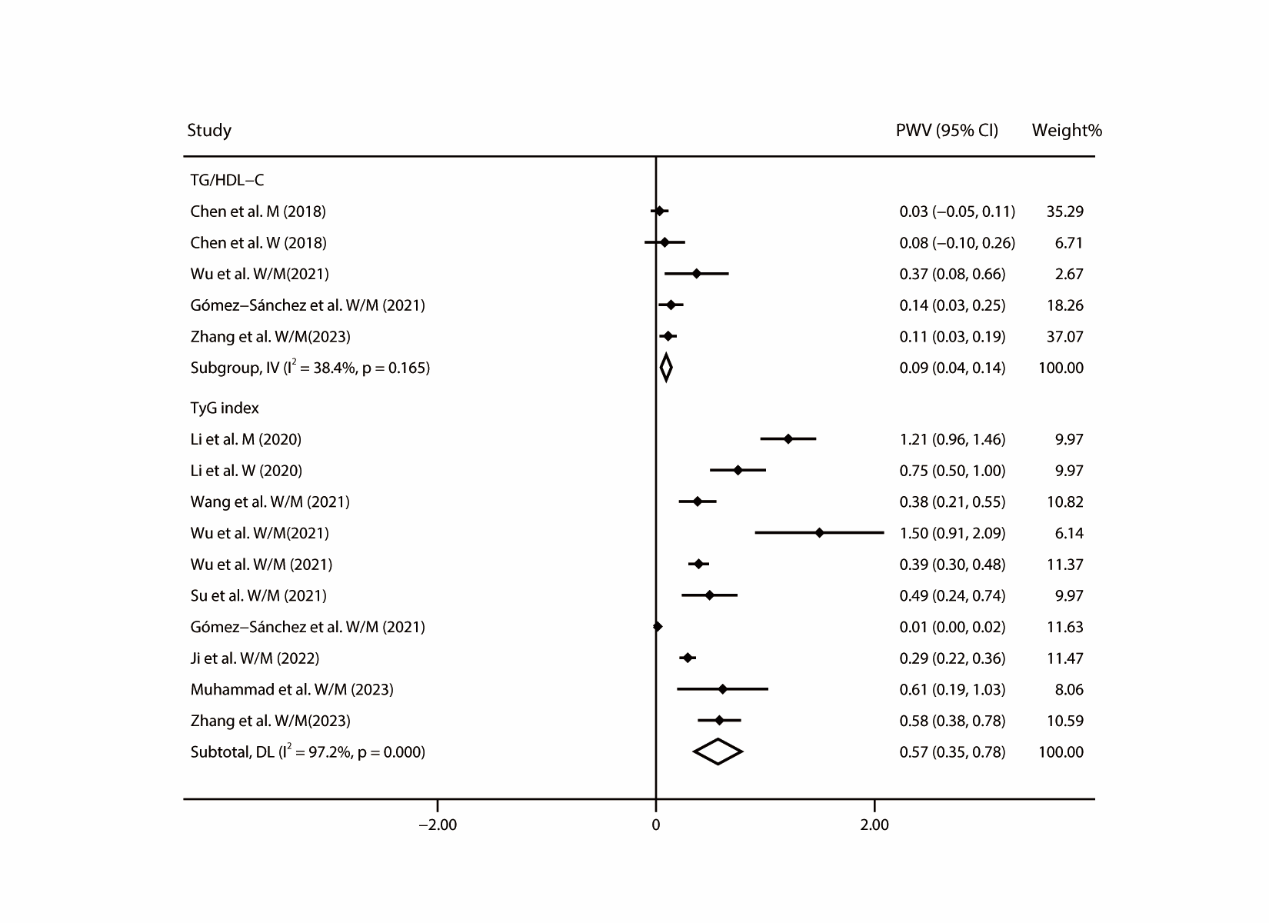


**Supplemental Figure 5.** Forest plot of pooled association between TG/HDL-C and TyG index (per 1 unit increment) and baPWV levels (m/s).
